# The CSP-Based New Features Plus Non-Convex Log Sparse Feature Selection for Motor Imagery EEG Classification

**DOI:** 10.3390/s20174749

**Published:** 2020-08-22

**Authors:** Shaorong Zhang, Zhibin Zhu, Benxin Zhang, Bao Feng, Tianyou Yu, Zhi Li

**Affiliations:** 1 School of Electronic Engineering and Automation, Guilin University of Electronic Technology, Guilin 541004, China; zsrong@guat.edu.cn (S.Z.); bxzhang@guet.edu.cn (B.Z.); cclizhi@guet.edu.cn (Z.L.); 2 School of Electronic Information and Automation, Guilin University of Aerospace Technology, Guilin 541004, China; fengbao@guat.edu.cn; 3 School of Mathematics and Computational Science, Guangxi Colleges and Universities Key Laboratory of Data Analysis and Computation, Guilin University of Electronic Technology, Guilin 541004, China; 4 School of Automation Science and Engineering, South China University of Technology, Guangzhou 510000, China; auyuty@scut.edu.cn

**Keywords:** brain-computer interface (BCI), electroencephalogram (EEG), motor imagery, common spatial pattern (CSP), feature extraction, feature selection

## Abstract

The common spatial pattern (CSP) is a very effective feature extraction method in motor imagery based brain computer interface (BCI), but its performance depends on the selection of the optimal frequency band. Although a lot of research works have been proposed to improve CSP, most of these works have the problems of large computation costs and long feature extraction time. To this end, three new feature extraction methods based on CSP and a new feature selection method based on non-convex log regularization are proposed in this paper. Firstly, EEG signals are spatially filtered by CSP, and then three new feature extraction methods are proposed. We called them CSP-wavelet, CSP-WPD and CSP-FB, respectively. For CSP-Wavelet and CSP-WPD, the discrete wavelet transform (DWT) or wavelet packet decomposition (WPD) is used to decompose the spatially filtered signals, and then the energy and standard deviation of the wavelet coefficients are extracted as features. For CSP-FB, the spatially filtered signals are filtered into multiple bands by a filter bank (FB), and then the logarithm of variances of each band are extracted as features. Secondly, a sparse optimization method regularized with a non-convex log function is proposed for the feature selection, which we called LOG, and an optimization algorithm for LOG is given. Finally, ensemble learning is used for secondary feature selection and classification model construction. Combing feature extraction and feature selection methods, a total of three new EEG decoding methods are obtained, namely CSP-Wavelet+LOG, CSP-WPD+LOG, and CSP-FB+LOG. Four public motor imagery datasets are used to verify the performance of the proposed methods. Compared to existing methods, the proposed methods achieved the highest average classification accuracy of 88.86, 83.40, 81.53, and 80.83 in datasets 1–4, respectively. The feature extraction time of CSP-FB is the shortest. The experimental results show that the proposed methods can effectively improve the classification accuracy and reduce the feature extraction time. With comprehensive consideration of classification accuracy and feature extraction time, CSP-FB+LOG has the best performance and can be used for the real-time BCI system.

## 1. Introduction

The brain computer interface (BCI) converts the brain signals into external device control commands, which establishes a new channel for humans to directly interact with the external environment [1]. This technique is particularly useful for patients with motor disability and upper body paralysis [2]. Of course, BCI can also be used for healthy people, such as games or robot control [3]. Among various brain signals, the scalp electroencephalogram (EEG) is easy to obtain. With low cost and high time resolution, EEG is widely used in BCI [4]. Motor imagery is a spontaneously generated EEG signal, which does not require external stimulation. It is particularly suitable for patient rehabilitation training and motor control. However, the EEG signal is very weak, with a low signal-to-noise ratio and space blurred [5]. It is very difficult to extract stable and discriminative features. Therefore, feature extraction has always been a hotspot in the study of motor imagery based BCI. In addition, feature selection can reduce feature dimension and noise interference, the selected features are more stable and discriminative. Therefore, research on feature selection is also very important.

Commonly used feature extraction methods include the autoregressive model [6], wavelet features [7], band power [8], and common spatial pattern (CSP) [9]. CSP can effectively extract the features of event-related synchronization (ERS) and event-related desynchronization (ERD) in the motor imagery signals, so it has been widely used in BCI [10]. However, the performance of CSP depends to a large extent on the selection of the filtering frequency band, and the optimal frequency band is typically subject-specific, which is difficult to select manually [11]. There is a lot of research work on the frequency band selection, which is mainly divided into four categories. The first type of method, CSP combined with time-frequency analysis methods. Based on orthogonal empirical mode decomposition (OEMD), FIR filter, and the CSP algorithm, Li et al. [12] proposed a novel feature extraction method. Lin et al. [13] used wavelet-CSP algorithm to recognize driving action. Robinson et al. [14] used the wavelet-CSP algorithm to classify fast and slow hand movements. Feng et al. [15] proposed a feature extraction algorithm based on CSP and wavelet packet for motor imagery EEG signals, and Yang et al. [16] proposed subject-based feature extraction using the fisher WPD-CSP method. The second type of method, the spatial spectrum filter is optimized simultaneously. For example, the common spatio-spectral pattern algorithm (CSSP) is proposed by Lemm et al. [17], the common sparse spectral spatial pattern algorithm (CSSSP) is proposed by Dornhege et al. [18], and a new discriminant filter bank common spatial patterns (DFBCSP) is proposed by Hiroshi et al. [19]. The third type of method, the original EEG signals are filtered into multiple frequency bands, then the CSP features are extracted in each band, and finally the features of the optimal frequency band are selected for classification. There are many research works in this area, such as SBCSP [20], FBCSP [11], DFBCSP [21], SWDCSP [22], SFBCSP [23], and SBLFB [24]. The fourth type of method, the intelligent optimization method, is used to select the optimal frequency band. The multiple fixed frequency bands used in the third method are determined by human subjective experience, so the obtained frequency band may not be optimal, while the intelligent optimization algorithm can select a frequency band of any length. Wei et al. [25] used binary particle swarm optimization for frequency band selection in motor imagery-based brain-computer interfaces. Kumar et al. [26] proposed three methods to optimize the temporal filter parameters, including particle swarm optimization (PSO), genetic algorithm (GA), and artificial bee colony (ABC). Rivero et al. [27] used genetic algorithms and k-nearest neighbor for automatic frequency band selection. The first method uses the time-frequency analysis to obtain frequency information. It needs to decompose the EEG signals of each channel, which requires a large amount of calculation and is time-consuming, especially for the wavelet packet decomposition. The second method is difficult to solve and easy to get a local solution. The EEG signals are filtered into multiple sub-bands in the third method, which is very computationally intensive. The disadvantage of the fourth method is that it requires a long time for model training. Recently, the application of deep learning in motor imaging classification has become more and more widespread [28]. Tang et al. [29] used conditional empirical mode decomposition (CEMD) and one-dimensional multi-scale convolutional neural network (1DMSCNN) to recognize motor imagery EEG signals. Cheng et al. [30] classified EEG emotions by deep forest. However, features extracted by deep learning are abstract and difficult to understand [31]. In addition, compared with traditional machine learning methods, deep learning has no obvious advantages [32].

The existing feature selection methods are mainly divided into three categories: filter, wrapper, and embedded [33]. The filter method uses independent evaluation criteria, and the feature selection process of which has nothing to do with subsequent classifiers. Koprinska et al. [34] proposed five feature selection methods for the brain computer interface, including information gain ranking, correlation-based feature selection, relief, consistency-based feature selection, and 1R ranking. Experimental results show that the top three feature selectors in terms of classification accuracy were correlation-based feature selection, information gain and 1R ranking. Mutual information and its one-versus rest multi-class extension were used to select optimal spatial-temporal features in [35]. Li et al. [36] combined the Fisher score and classifier-dependent structure to implement the feature selection. Based on the descending sort on all Fisher score values, the wrapper model with support vector machine (SVM) and graph regularized extreme learning machine (GELM) were applied, and a 10-fold cross validation scheme was used to select the generalized features based on the training set. Mehmood et al. [37] selected the optimal EEG features using a balanced one-way ANOVA after calculating the Hjorth parameters for different frequency ranges. Features selected by this statistical method outperformed univariate and multivariate features. The optimal features were further processed for emotion classification using SVM, k-nearest neighbor (k-NN), linear discriminant analysis (LDA), naive Bayes, random forest, deep learning, and four ensembles methods (bagging, boosting, stacking, and voting). The maximum of average distance between events and non-events was used to select optimal EEG features in [38]. The filter method has certain advantages, such as low computational cost, but it does not consider the correlation between features and is independent of the classifier, so the classification accuracy is not high. The wrapper method uses the performance of classifier as the evaluation criterion of feature selection. An efficient feature selection method was proposed in [39]. The least angle regression (LARS) was used for properly ranking each feature, and then an efficient leave-one-out (LOO) estimation based on the PRESS statistic was used to choose the most relevant features. In [40], the genetic algorithm was used to select the EEG signal features. The fitness function used in the genetic algorithm was EEG signal classification error calculated using LDA classifier. Rakshit et al. [41] employed ABC cluster algorithm to reduce the features for motor imagery EEG data. Baig et al. [42] proposed a new hybrid method to select features. A differential evolution (DE) optimization algorithm was used to search the feature space to generate the optimal feature subset, and with performance evaluated by the SVM classifier. Liu et al. [43] proposed a method of combining the firefly algorithm and learning automata (LA) to optimize feature selection for motor imagery EEG. The learning automata was used as a tool of parameter optimization to avoid getting the local optimum. The wrapper method needs to train and test the classifier when evaluating each candidate feature subset, which is computationally expensive and tends to overfitting. The embedded method integrates feature selection with the training process of the classifier, and simultaneously performs feature selection and classification. Therefore, the embedded feature selection method has been widely used in recent years. Miao et al. [44] used LASSO to select the important space-frequency-time feature components of motor imagery. The minimum-redundancy and maximum-relevance (mRMR) and LASSO were used for feature selection in [45]. In both feature selection methods, the first three features were selected. Then, the common features between mRMR and LASSO regularization are selected to train the classification model. Zhang et al. [46] proposed a novel algorithm, namely the temporally constrained sparse group spatial pattern (TSGSP), which was modeled by combining the sparse group LASSO and fused LASSO penalties. The features with different filter bands and time window combinations were optimized and selected. Wang et al. [47] used the sparse group LASSO to simultaneously perform feature selection and channel selection on the motor imagery signal. Jiao et al. [48] proposed a sparse group LASSO representation model for transfer learning, the group LASSO selected subjects, and LASSO selected sample data. The above sparse optimization methods are convex optimization models. Although they have achieved good results, many applications have shown that non-convex sparse optimization methods can obtain better performance [49]. For example, LASSO has a bias problem, which would result in significantly biased estimates, and cannot achieve reliable recovery with the least observations [50].

Aiming to resolve the problem of large calculation and time-consumption of Wavelet-CSP [13,14], WPD-CSP [15,16], and FBCSP [11] methods, we have proposed three new feature extraction methods, namely CSP-Wavelet, CSP-WPD, and CSP-FB method. Firstly, the original EEG signals are pre-processed, including time window selection and band-pass filtering. Then, CSP transform is performed. For CSP-Wavelet, discrete wavelet transform (DWT) is used to decompose the spatially filtered signals, and then the energy and standard deviation of the wavelet coefficients are extracted as features. For CSP-WPD, the wavelet packet decomposition (WPD) is used to decompose the spatially filtered signals. Similar to CSP-Wavelet, the energy and standard deviation of the wavelet coefficients are extracted as features. For CSP-FB, the spatially filtered signals are filtered into multiple frequency bands by a filter bank (FB), and then the logarithm of variances of each band are extracted as features. In order to solve the bias problem of LASSO, a new feature selection method is proposed. A non-convex function is used to sparsely constrain feature weights. Since the non-convex function is a log function, we call this method LOG. In addition, in order to further optimize feature selection and enhance the robustness of the classification model, an ensemble learning method is proposed for secondary feature selection and the construction of multiple classification models. Fisher linear discriminant analysis (FLDA) is used for classification. Combining feature extraction with feature selection methods, we obtained three EEG signals decoding methods, namely CSP-Wavelet+LOG, CSP-WPD+LOG and CSP-FB+LOG. Experimental results show that the classification performances of three newly proposed methods are better than CSP, Wavelet-CSP, WPD-CSP, SFBCSP and SBLFB methods. In terms of feature extraction time, the proposed methods are much less than Wavelet-CSP, WPD-CSP, SFBCSP, and SBLFB methods.

The main contributions of this paper include three aspects. Firstly, we proposed three new feature selection methods based on CSP. These three methods can effectively improve the classification performance of CSP while reducing the feature extraction time. Secondly, we propose a new feature selection method. This method is a non-convex sparse optimization method, which can effectively solve the bias problem of LASSO and select more discriminative features. Thirdly, we use ensemble learning for secondary feature selection and classification model construction, which makes the EEG decoding method more robust and stable.

The content of this paper is organized as follows. Section 2 introduces experimental data, traditional CSP feature extraction method, three new feature extraction methods, a new feature selection method, and secondary feature selection and classification model construction using ensemble learning. The experimental results are showed in Section 3. Section 4 further discusses and analyzes the experimental results. The conclusion is provided in Section 5.

## 2. Materials and Methods

### 2.1. EEG Data Description

Four public motor imagery EEG datasets are briefly described as follow. For detailed information, please refer to related literature or website.

Dataset 1: data set I of BCI competition IV (2008) [51]. This dataset has a total of 59 channels with a sampling rate of 100 Hz. There are three types of motor imagery tasks, including left hand, right hand, and right foot. Seven subjects (1a, 1b, 1c, 1d, 1e, 1f, 1g) selected two of them to be performed. In this paper, the calibration data of this dataset are used for classification, which include two runs with 100 single trials for each run. The first run was selected as the training set and the second run was selected as the test set. For detailed information, please refer to the following website: http://www.bbci.de/competition/IV/.

Dataset 2: data set IIa of BCI competition IV (2008) [52]. This dataset has a total of 22 channels with a sampling rate of 250Hz. Nine subjects (A01, A02, …, and A09) performed four types of motor imagery tasks, including left hand, right hand, foot, and tongue. There are two sessions in this dataset, and each session was consisted of 6 runs with 48 trials (12 trials for each class) for each run. In this paper, the first session was selected as the training set and the second session was selected as the test set. The training and test sets of each subject were 72 trials. According to the practice of reference [53], four types of tasks are arranged and combined to obtain multiple binary classification problems, that is, $C_{4}^{2}=6$ groups of binary classification. Therefore, a total of 9 × 6 = 54 data subsets are obtained. The left hand, right hand, foot, and tongue motor imagery tasks were represented by letters L, R, F and T, respectively. A01T -LR indicated that the subject A01T performed left hand and right hand motor imagery tasks. For additional information, please refer to the following website: http://www.bbci.de/competition/IV/.

Dataset 3: data set IIb of BCI competition IV (2008) [24]. This dataset has a total of 3 channels with a sampling rate of 250Hz. Nine subjects (B01, B02, …, and B09) performed two types of motor imagery tasks, including left hand and right hand. There are five sessions in this dataset. However, only the third training session (B0103T, B0203T, …, B0903T) is used in this paper [24]. This session is consisted of 160 trials, and half for each class. 80 trials are used for training set, and the other 80 trials are used for test set. For additional information, please refer to the following website: http://www.bbci.de/competition/IV/.

Dataset 4: data set provided by David Steyrl (2016) [54]. This dataset has a total of 15 channels with a sampling rate of 512 Hz. Fourteen subjects performed two types of motor imagery tasks, including right hand and foot. The data of each subject were divided into two parts. The first part (runs 1–5) was used for train set, whereas the second part (runs 6–8) was used for test set, and each run was consisted of 20 trials (10 trials for each class). Therefore, the training and test sets are 100 and 60 trials, respectively. The original signals are downsampled with a sampling rate of 256 Hz. For more information, please refer to the following website: http://bnci-horizon-2020.eu/database/data-sets.

**Figure 1:**
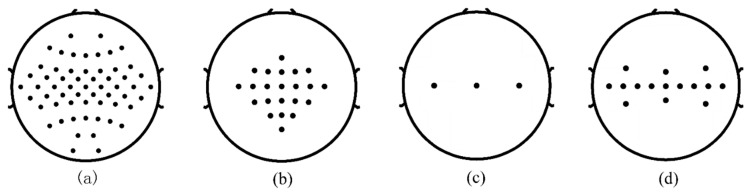
Distribution of electrodes on the scalp for all datasets. (**a**) dataset 1, (**b**) dataset 2, (**c**) dataset 3. (**d**) dataset 4.

All datasets are scalp EEG signals, which are recorded by multiple electrode sensors placed on the scalp. Figure 1 shows the distribution of electrodes on the scalp for the four datasets. We focus on the signal processing and pattern recognition of electrode sensor signals in this paper.

### 2.2. The Processing Flow of the Proposed Method

**Figure 2:**
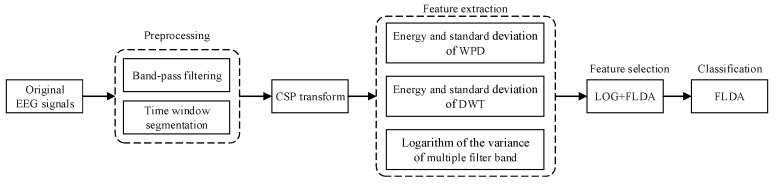
The processing flow of the proposed method.

Figure 2 is a flowchart of the overall processing of the proposed method. It mainly includes preprocessing, CSP transformation, feature extraction, feature selection, and classification. Each part will be discussed in detail in the following content.

### 2.3. Data Preprocessing

(1) A 6th order Butterworth filter is used to perform 8–30 Hz band-pass filtering on the EEG signals of each channel, which filters out the EEG components that are not related to motor imagery. Butterworth filters are often used in EEG band-pass filtering [53], we are consistent with the practice of most literatures. Motor imagery can cause ERS and ERD phenomena, that is, power changes in specific frequency bands of EEG signals, specifically mu (8–12 Hz) and beta (18–26 Hz) rhythm [2]. Therefore, the 8–30 Hz band-pass filter is usually used to filter the motor imagery signals [55].
(2) Extracting single trial data. The time window of dataset 1 is 0.5–3.5 s, and the other datasets are 0.5–2.5 s, where 0 s is the time when the motor imagery task starts. The time window of datasets 2–4 is different from that of dataset 1. This is because the sampling rate of datasets 2–4 is relatively high. Choosing the time window from 0.5 s to 2.5 s can reduce the amount of data and thus reduce the amount of calculation.

### 2.4. Feature Extraction

#### 2.4.1. CSP Transformation

For the binary classification problem, CSP looks for a set of spatial filters to maximize the variance of the band-pass filtered EEG signals in one class while minimizing the other class. The spatial filter w is calculated by simultaneous diagonalization of sample covariance matrix from both classes, details as follows:

(1) $$J(w)=\frac{w^{T}{\bar{C}}_{1}w}{w^{T}{\bar{C}}_{2}w}$$

where T denotes transpose. ${\bar{C}}_{1}$ and ${\bar{C}}_{2}$ represent the average covariance matrix of two types of tasks, respectively, which are defined as follows:

(2) $${\bar{C}}_{k}=\frac{1}{N_{k}}\sum_{n=1}^{N_{k}}\frac{D_{\left(k,n\right)}D_{\left(k,n\right)}^{T}}{trace\left(D_{\left(k,n\right)}D_{\left(k,n\right)}^{T}\right)},\;k=1,2$$

where trace(·) denotes the solution of the matrix trace. $N_{k}$ represents the number of samples of the kth task, that is, the number of single trial data. $D_{\left(k,n\right)}\in {\mathbb{R}}^{C\times K}$ represents the nth trial data of the kth task, where C represents the total number of EEG signal channels, and K represents the number of samples of each channel.

Formula (1) can be transformed into the following generalized eigenvalue problem [55].

(3) $${\bar{C}}_{2}^{-1}{\bar{C}}_{1}w=\lambda w$$

The spatial filters are then the eigenvectors of $M=\;{\bar{C}}_{2}^{-1}{\bar{C}}_{1}\;$. The M is arranged in descending order of eigenvalues to obtain $\tilde{M}$, and the feature vectors of the first m columns and the last m columns of $\tilde{M}$ are usually taken as the final spatial filter, which is denoted as W. In all experiments in this paper, m is set to 3. For single trial data D, its spatial projection signal is:

(4) $$Z=W^{T}D$$

The traditional CSP feature extraction method extracts the logarithm of the variances of spatially filtered signals as features, details as follows:

(5) $$f_{p}=\log\left(\frac{\mathrm{var}\left(Z_{p}\right)}{\sum_{i=1}^{2m}\mathrm{var}\left(Z_{i}\right)}\right),p=1,2,\cdots ,2m$$

where var(·) represents the solution of the variance. Finally, the feature vector of the single trial data can be obtained by calculating the formula (5), that is $x=[f_{1},f_{2},\cdots ,f_{2m}]$.

#### 2.4.2. New Feature Extraction Methods

##### CSP-Wavelet

**Figure 3:**
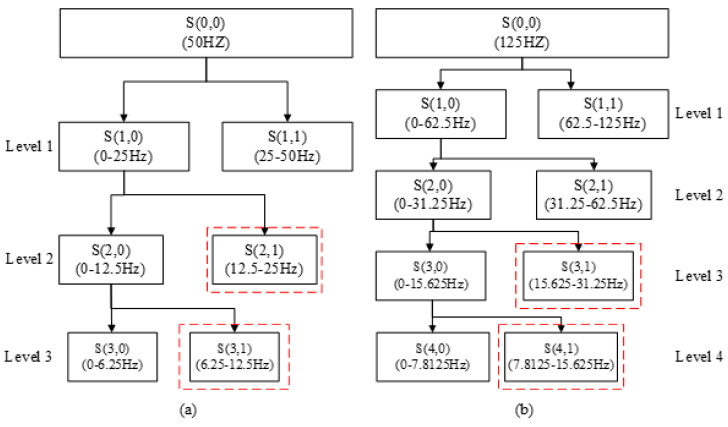
Wavelet decomposition with different sampling rates. (**a**) The sampling rate is 100 Hz and the number of decomposition layers is 3. (**b**) The sampling rate is 250 Hz and the number of decomposition layers is 4.

After spatial filtering, we can get Z in Section 2.4.1. Discrete wavelet transform is performed on each channel of Z. The derivation of the wavelet decomposition formula is described in detail in [14], interested readers can refer to literature [14]. After the wavelet decomposition, the frequency sub-bands related to motor imagery are selected, and the energy and standard deviation of the wavelet coefficients of the selected sub-bands are extracted as features. The wavelet base is db4. In order to select sub-bands related to motor imagery, we need to combine the sampling rate of the dataset to select the appropriate number of wavelet decomposition layers. For dataset 1 (the sampling rate is 100 Hz), the number of decomposition layers is 3 in this paper. For datasets 2–4 (the sampling rate is 250 and 256 Hz, respectively), the number of decomposition layers is 4. The selection of the number of decomposition layers will be discussed in detail in the discussion section. Figure 3 shows the process of wavelet decomposition with different sampling rates. The sampling rate of 256 Hz and 250 Hz are very close, so we only consider the decomposition process of 250 Hz, and the selected sub-bands of 256 Hz and 250 Hz are the same. Wavelet coefficients with sub-band frequencies in the range of 8–30 Hz are selected and used for feature extraction. The selected sub-bands are marked by the red dotted frame in Figure 3.

The energy of the wavelet coefficients of the selected sub-bands is calculated as follows:

(6) $$e_{i}=\sum_{j=1}^{N}{\left|D_{ij}\right|}^{2},\;i=1,2,\cdots ,B$$

where B represents the number of selected sub-bands, N represents the number of the wavelet coefficients, and $D_{ij}$ represents the jth wavelet coefficient of the ith sub-band.

The standard deviation of the wavelet coefficients of the selected sub-bands is calculated as follows:

(7) $$s_{i}={\left(\frac{1}{N-1}\sum_{j=1}^{N}{\left(D_{ij}-\mu_{i}\right)}^{2}\right)}^{1/2},\;i=1,2,\cdots ,B$$

the meaning of B, N and $D_{ij}$ is consistent with formula (6) and $\mu_{i}$ represents the average value of the wavelet coefficients of the ith sub-band. Finally, we can get the feature vector for the CSP-Wavelet feature extraction method as follows:

(8) $$x_{DWT}=[\underset{channel\;1}{\underset{︸}{e_{1}^{1},s_{1}^{1},e_{2}^{1},s_{2}^{1},\cdots ,e_{B}^{1},s_{B}^{1}}};\underset{channel\;2}{\underset{︸}{e_{1}^{2},s_{1}^{2},e_{2}^{2},s_{2}^{2},\cdots ,e_{B}^{2},s_{B}^{2}}};\cdots \cdots ;\underset{channel\;2m}{\underset{︸}{e_{1}^{2m},s_{1}^{2m},e_{2}^{2m},s_{2}^{2m},\cdots ,e_{B}^{2m},s_{B}^{2m}}}]$$

where $e_{i}^{c}$ and $s_{i}^{c}$ represent energy and standard deviation of the ith sub-band of the cth channel of Z, respectively.

##### CSP-WPD

**Figure 4:**
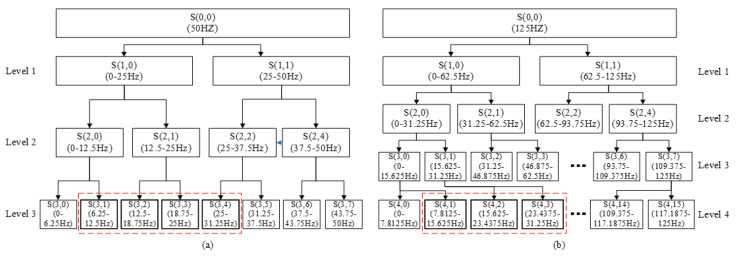
Wavelet packet decomposition with different sampling rates. (**a**) The sampling rate is 100 Hz and the number of decomposition layers is 3. (**b**) The sampling rate is 250 Hz and the number of decomposition layers is 4.

Similar to CSP-Wavelet, Wavelet packet decomposition is performed on each channel of Z. The derivation of the wavelet packet decomposition formula is described in detail in [56], interested readers can refer to literature [56]. The energy and standard deviation of the wavelet coefficients of the selected sub-bands are extracted as features. The wavelet base and the number of decomposition layers are the same as CSP-Wavelet. Figure 4 shows the process of wavelet packet decomposition with different sampling rates. Wavelet coefficients with sub-band frequencies in the range of 8–30 Hz are selected and used for feature extraction. Similar to CSP-Wavelet, the selected sub-bands of 256 Hz and 250 Hz are the same. The selected sub-bands are marked by the red dotted frame in Figure 4.

The calculation of the energy and standard deviation are the same as CSP-Wavelet, so the final feature vector form is similar, details as follow:

(9) $$x_{WPD}=[\underset{channel\;1}{\underset{︸}{e_{1}^{1},s_{1}^{1},e_{2}^{1},s_{2}^{1},\cdots ,e_{B^{\prime }}^{1},s_{B^{\prime }}^{1}}};\underset{channel\;2}{\underset{︸}{e_{1}^{2},s_{1}^{2},e_{2}^{2},s_{2}^{2},\cdots ,e_{B^{\prime }}^{2},s_{B^{\prime }}^{2}}};\cdots \cdots ;\underset{channel\;2m}{\underset{︸}{e_{1}^{2m},s_{1}^{2m},e_{2}^{2m},s_{2}^{2m},\cdots ,e_{B^{\prime }}^{2m},s_{B^{\prime }}^{2m}}}]$$

where $B^{\prime }$ represents the number of the selected sub-bands of CSP-WPD.

##### CSP-FB

After spatial filtering, the signals of each channel of Z are filtered into 10 sub-bands with bandwidth of 4 Hz and the overlap rate of 2 Hz in the range of 8–30 Hz. A 6th order Butterworth filter is used. Then, the logarithm of the variances of each sub-band are extracted as features. So, the final feature vector is

(10) $$x_{FB}=[\underset{filter\;band\;1}{\underset{︸}{f_{1}^{1},f_{2}^{1},\cdots ,f_{2m}^{1}}};\underset{filter\;band\;2}{\underset{︸}{f_{1}^{2},f_{2}^{2},\cdots ,f_{2m}^{2}}};\cdots \cdots ;\underset{filter\;band\;B^{\prime \prime }}{\underset{︸}{f_{1}^{B^{\prime \prime }},f_{2}^{B^{\prime \prime }},\cdots ,f_{2m}^{B^{\prime \prime }}}}]$$

where $B^{\prime \prime }$ represents the number of the filter bands.

### 2.5. Feature Selection

After feature extracted, we can get a sample feature matrix $X={\left(x_{1},x_{2},\ldots ,x_{N}\right)}^{T}$, where $X\in {\mathbb{R}}^{N\times P}$, N is the total number of feature samples, P is the dimension of feature sample, $x_{i}\in \mathbb{R}{\;}^{P},\;i\in (1,2,\;\ldots ,N)$ represents the ith feature sample (feature vector). According to different feature extraction methods, x can be $x_{DWT}$, $x_{WPD}$, or $x_{FB}$.

The feature vector obtained by the feature extraction method usually contains redundant information. The redundant features not only increase the complexity of the classification model and model training time, but also easily lead to overfitting. Therefore, feature selection is required to remove redundant features and improve the classification accuracy. LASSO [57] is often used for feature selection, and its mathematical model is as follows:

(11) $$\underset{w}{\min}\frac{1}{2}{\left\|y-Xw\right\|}_{2}^{2}+\lambda {\left\|w\right\|}_{1}$$

where λ>0 is the regularization parameter, w is feature weight, ${\left\|w\right\|}_{1}=\sum_{i=1}^{P}\left|w_{i}\right|$, and $w_{i}$ is the ith element of w, $y={\left(y_{1},y_{2},\ldots ,y_{N}\right)}^{T}$ are the sample labels and $y_{i}\in \{-1,1\}$. Although LASSO is widely used and works well, features selected by LASSO are usually too sparse and scattered throughout the feature space. When the feature dimension is much larger than the sample size, which is very common in EEG signal decoding, the selected results are unstable [58]. In addition, LASSO has a bias problem, which would result in significantly biased estimates, and cannot achieve reliable recovery with the least observations [59].

In order to ameliorate the bias problem of LASSO, we proposed a non-convex sparse optimization method for feature selection. The mathematical model can be described as follows:

(12) $$\underset{w}{\min}\frac{1}{2}{\left\|y-Xw\right\|}_{2}^{2}+\lambda \log(1+\frac{{\left\|w\right\|}_{1}}{a})$$

where a>0 is the scale parameters, a is set to 0.001 in this paper. This concave LOG function has the better ability to encourage the sparsity than $l_{1}$-norm and penalizes all elements non-uniformly [60]. In order to solve the minimization problem (12), many efficient algorithms are proposed, such as proximal algorithms [61] or the alternating direction of multipliers method [62]. The proximal splitting algorithms including iterative shrinkage thresholding [63] are popular methods for solving (11) and (12). Using proximal gradient methods has many advantages than the other methods. They can deal with general conditions, for functions which are non-convex, or non-smooth and convex. Those algorithms have simple forms and it is easy to derive and implement. In particular, they can be used in large scale problems.

So, in this paper we use the iterative log thresholding [64] to solve (12). It has only two basic steps which are iterated until convergence: (i) Gradient step. Define an intermediate point $v_{t}$ at the tth step by taking a gradient step with respect to the differentiable term

(13) $$v_{t}=w_{t}-1/\gamma \left(X^{T}\left(Xw_{t}-y\right)\right)$$

(ii) Proximal operator step. Evaluate the proximal operator of the non-convex log function at the intermediate point $v_{t}$

(14) $$w_{t+1}={\mathrm{prox}}_{\gamma \log}\left(v_{t}\right)={\mathrm{prox}}_{\gamma \log}\left(w_{t}-1/\gamma \left(X^{T}\left(Xw_{t}-y\right)\right)\right)$$

where $\gamma ={\left\|X^{T}X\right\|}_{2}$ and ${\mathrm{prox}}_{\gamma \log}\left(v_{t}\right)$ is the proximal operator of log regularization function, which is defined as

(15) $$w_{t+1}={\mathrm{prox}}_{\gamma \log}\left(v_{t}\right)=\arg\underset{w}{\min}\lambda \log(1+\frac{{\left\|w\right\|}_{1}}{a})+\frac{\gamma }{2}{\left\|w-v_{t}\right\|}_{2}^{2}$$

From the paper [65], we know that (15) has the explicit solution. Therefore, $w_{t+1}$ is given by the log function’s proximal operator:

(16) $$w_{t+1}=sign\left(v_{t}\right)/2\left(\left|v_{t}\right|-a+\sqrt{{\left(a-\left|v_{t}\right|\right)}^{2}+4\times \max\left(a\left|v_{t}\right|-\lambda /\gamma ,0\right)}\right)$$

where $sign\left(v_{t}\right)$ is the algebraic sign of $v_{t}$. Hence, the detailed iteration steps for solving (12) can be expressed as

(17) $$\left\{ \begin{array}{l} v_{t}=w_{t}-1/\gamma \left(X^{T}\left(Xw_{t}-y\right)\right)\\ w_{t+1}=sign\left(v_{t}\right)/2\left(\left|v_{t}\right|-a+\sqrt{{\left(a-\left|v_{t}\right|\right)}^{2}+4\times \max\left(a\left|v_{t}\right|-\lambda /\gamma ,0\right)}\right) \end{array}\right.$$

We can see the iteration scheme (17) is easy to implement and only involved the matrix vector multiplication. Also, every step has a closed form solution. It is suitable for the large scale problems. At last, the convergence of (17) is established in [64], and we refer the interested readers to [64] for more details.

### 2.6. Secondary Feature Selection and Classification Model Construction

**Figure 5:**
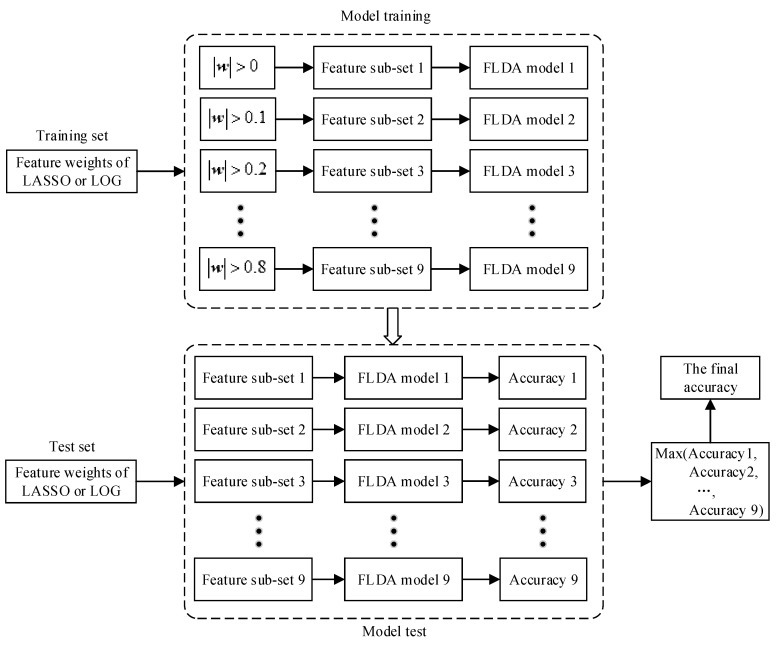
The processing flow of secondary feature selection and classification.

In order to select more effective features and construct a more robust classification model, we propose an ensemble learning method for secondary feature selection and the construction of multiple classification models. The overall processing flow is shown in Figure 5, where |w| represents the absolute value of w. In Section 2.5, we can obtain feature weights after feature selection performed by LASSO or LOG method. We further select features by setting a series of weight thresholds. The candidate threshold parameters are: [0, 0.1, ..., 0.8]. The features whose weight is bigger than the set threshold will be selected.

During the training phase, different thresholds will get different feature subsets, and feature subsets are trained to get different classification models. In this paper, we use FLDA as the classifier, so we get multiple FLDA classification models. During the test phase, we use the rules in the training phase to select a subset of features, and then use the classification models obtained in the training phase for classification. Because there are multiple classification models, we can get multiple classification accuracy. We take the maximum of these classification accuracy as the final classification accuracy. It is worth noting that if the feature subset is empty, the classification accuracy is directly set to 0.

Traditional machine learning methods use cross-validation to select the optimal feature subset and classification model in the training phase, and then use the obtained classification model for classification in the testing phase. However, our method trains multiple models and selects the maximum value of them as the final accuracy. This is where our work differs from previous research work. EEG signals have strong randomness and non-stationarity, and are also easily affected by the surrounding environment and noise during the collection process. The optimal feature subset and classification model selected in the training phase may not be optimal in the test set when they are interfered by noise. Different data samples suffer from different interferences. When classifying, they may get the best classification results in different feature subsets and classification models. We choose the maximum value of multiple classification models as the final accuracy, which has a certain degree of anti-interference and can increase the stability and robustness of the EEG decoding model.

## 3. Results

### 3.1. Compared Methods and Parameter Settings

In this paper, we use classification accuracy as the evaluation criterion. The classification accuracy is equal to the number of corrected classifications divided by the total number of test sets. FLDA is used for classification. For all methods, except for SFBCSP and SBLFB methods, the original EEG signals are filtered by 8–30 Hz band-pass filters. The compared methods are listed in Table 1, and the parameter setting will be introduced below.

The regularization parameters of LASSO and LOG are selected using 10-fold cross-validation and grid search method. The alternative set of hyperparameters is: $\lambda \in {[2}^{-5}{,2}^{-4.8},\ldots 2^{4.8}{,2}^{5}]$. The LASSO was implemented using the SLEP toolbox [66].

### 3.2. Experimental Results and Analysis

Table 2 shows the classification accuracy of various subjects in dataset 1 for each method. Except for CSP, the three proposed methods are significantly better than the compared methods. The CSP-FB+LOG method has achieved the highest average classification accuracy among the three proposed methods, and the highest classification accuracy in multiple subjects. Four CSP improvement methods, including Wavelet-CSP, WPD-CSP, SFBCSP, and SBLFB, have lower average accuracy than the traditional CSP.

There are 6 types of binary classification tasks in dataset 2 and a total of 54 subject-tasks. Table 3 shows the classification accuracy of various subjects in dataset 2 for each method. The three proposed methods are significantly better than the compared methods. The CSP-Wavelet+LOG method has achieved the highest average classification accuracy, followed by CSP-WPD+LOG and CSP-FB+LOG. The Wavelet-CSP and WPD-CSP methods are slightly better than CSP, but the SFBCSP and SBLFB methods are lower than CSP.

Table 4 shows the classification accuracy of various subjects in dataset 3 for each method. The CSP-FB+LOG methods are significantly better than the compared methods. CSP-WPD+LOG and CSP achieved the same average classification accuracy, and CSP-Wavelet+LOG was slightly lower than CSP. Other methods are lower than CSP.

Table 5 shows the classification accuracy of various subjects in dataset 4 for each method. The three proposed methods are significantly better than the compared methods. The CSP-WPD+LOG method has achieved the highest average classification accuracy among the three proposed methods, and the highest classification accuracy in multiple subjects. The Wavelet-CSP and WPD-CSP methods are better than CSP, but the SFBCSP and SBLFB methods are still lower than CSP.

**Figure 6:**
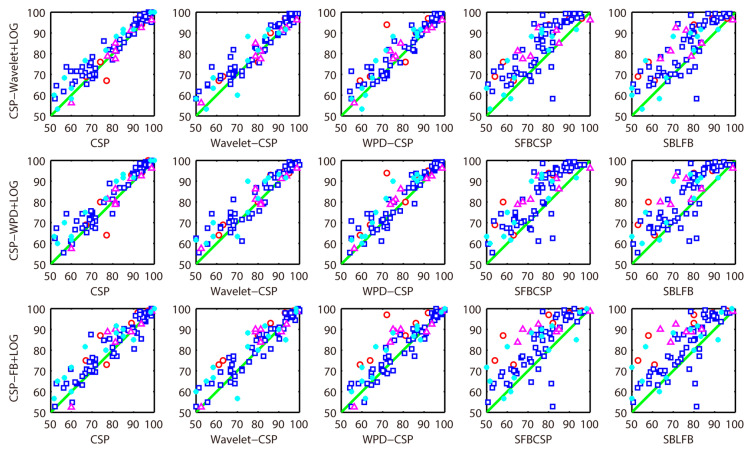
Classification accuracy comparison (All data).

In order to better demonstrate the superiority of the proposed methods, Figure 6 shows the classification accuracy of all methods in each subject. The red circle represents the classification accuracy of dataset 1 (seven subjects). The blue box represents the classification accuracy of dataset 2 (54 subjects). The cyan asterisk represents the classification accuracy of dataset 3 (nine subjects). The magenta triangle represents the classification accuracy of dataset 4 (14 subjects). Points above the diagonal indicate that the proposed methods are superior to the compared methods. From Figure 6, it can be seen that most of the points are above the diagonal, illustrating the superiority of the proposed methods.

**Figure 7:**
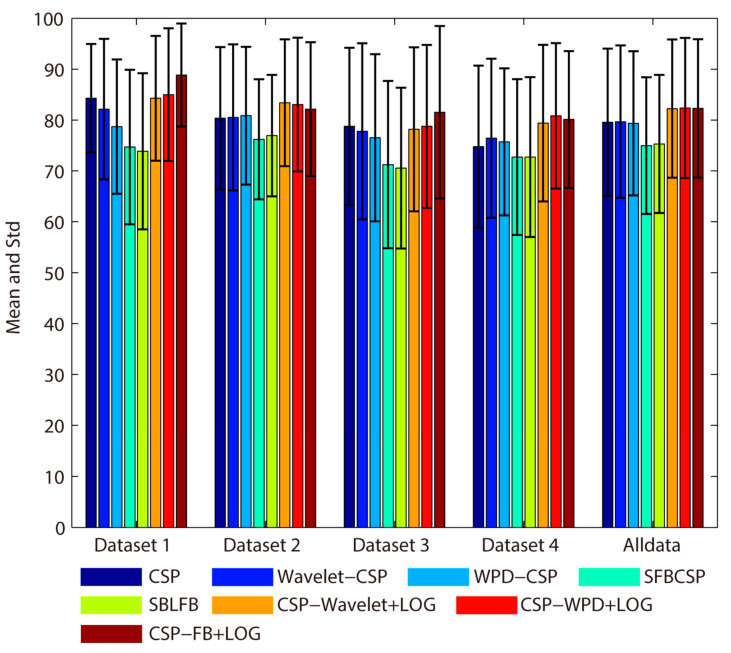
Average classification accuracy comparison (all data).

In order to show the overall classification effect of the proposed methods more intuitively, Figure 7 shows the average classification accuracy of each dataset and the total average classification accuracy of all data. From Figure 7, it can be seen that the proposed methods are significantly better than other methods. For all data, the average classification accuracy and standard deviation obtained by the CSP, Wavelet-CSP, WPD-CSP, SFBCSP, SBLFB, CSP-Wavelet+LOG, CSP-WPD+LOG, and CSP-FB+LOG methods are: 79.58 ± 14.47, 79.68 ± 14.98, 79.36 ± 14.17, 74.97 ± 13.42, 75.30 ± 13.58, 82.25 ± 13.57, 82.38 ± 13.77 and 82.3 ± 13.61, respectively. The CSP-WPD+LOG method achieves the highest average classification accuracy in all data. The CSP-Wavelet+LOG and CSP-FB+LOG are slightly lower than CSP-WPD+LOG. The Wavelet-CSP and WPD-CSP methods are slightly better than CSP. The SFBCSP and SBLFB methods are always lower than CSP in each dataset and all data.

In order to study the effectiveness of secondary feature selection, Table 6 and Table 7 show the classification results with secondary feature selection and without secondary feature selection. LASSO and LOG are used for feature selection, respectively. It can be seen from Table 6 and Table 7 that no matter which feature extraction method, the second feature selection has achieved better results. Especially for CSP-FB feature extraction method, the overall average classification accuracy is improved by 3.91% for LASSO, and 3.62% for LOG.

Comparing Table 6 and Table 7, we can analyze the performance of LASSO and LOG. First, we analyze the situation without secondary feature selection. For all data, except for CSP-WPD feature extraction method, the average classification accuracy of LOG is higher than LASSO. In the situation with secondary feature selection, LOG is better than LASSO. No matter whether secondary feature selection is performed, LOG is better to LASSO for CSP-Wavelet and CSP-FB feature extraction method. In summary, the LOG is superior to LASSO.

In addition to LASSO, our method is also compared with other three feature selection methods, and the corresponding results are showed in Table 8. Fisher score (F-score) [36] combined with FLDA classifier constitutes a wrapped feature selection method. The optimal feature subset is selected using 10-fold cross-validation. Genetic algorithm (GA) and binary particle swarm optimization (BPSO) can be referred to the literature [67], and the parameter settings and classifier of these two methods are consistent with literature [67]. After feature selection, FLDA is used for classification. For data set 1, when CSP-WPD is usd for feature extraction, the average classification accuracy of LOG is slightly lower than that of LASSO. For dataset 2, when CSP-FB is used for feature extraction, the average classification accuracy of LOG is slightly lower than that of LASSO. In other cases, LOG is better than LASSO. It can be seen from Table 8 that LOG has achieved the best classification effect, which is significantly better than other feature selection methods. In addition, F-score is better than GA and BPSO.

In order to study the effect of different classifiers on the performance of LOG, Table 9 shows the classification results of LOG using three classifiers. SVM is implemented using LIBSVM toolbox [68]. SVM uses the linear kernel function, and the parameter settings of SVM are set according to the toolbox default settings. Bayesian linear discriminant analysis (BLDA) [69] is an improvement of FLDA. The BLDA model parameters are automatically estimated from the training data. For CSP-Wavelet+LOG and CSP-WPD+LOG, the average accuracy (for all data) of FLDA is higher than that of SVM and BLDA. For CSP-FB+LOG, the average accuracy of FLDA is almost the same as that of SVM and BLDA. In general, FLDA is better than SVM and BLDA. Therefore, when selecting a classifier, FLDA is a better choice for the proposed methods in this paper.

In order to more comprehensively evaluate the effectiveness of our method, Table 10, Table 11 and Table 12 respectively show the classification results of our method and other existing methods in the three BCI data sets.

Table 10 shows the classification accuracy of the proposed methods and other resent methods for BCI Competition IV Dataset I. It can be seen from Table 10 that CSP-FB+LOG is second only to literature [73]. The average classification accuracy of CSP-WPD+LOG is lower than that of literatures [73,74]. CSP-Wavelet+LOG is not good but not bad either, rather the effect is mediocre. The literature [73] proposed a novel feature extraction method in which the hybrid features of the brain function based on the bilevel network are extracted. Minimum spanning tree (MST) based on EEG signal nodes in different motor imagery is constructed as the first network layer to solve the global network connectivity problem. In addition, the regional network in different movement patterns is constructed as the second network layer to determine the network characteristics, which is consistent with the correspondence between limb movement patterns and cerebral cortex in neurophysiology. Although literature [73] has achieved better results, it is stronger a priori both in terms of frequency bands and EEG electrodes used to perform the classification. Our method does not require any prior information.

Table 11 shows the classification accuracy of the proposed methods and other resent methods for BCI Competition IV Dataset IIa. It can be seen from Table 11 that CSP-Wavelet+LOG and CSP-WPD+LOG is second only to literature [80]. CSP-FB+LOG is slightly lower than the literatures [76,77]. Although the literature [80] has achieved good classification results, the literature [80] relies on the data of other subjects. Our method only uses data from the subjects themselves. Therefore, our method is more independent.

Table 12 shows the classification accuracy of the proposed methods and other resent methods for BCI Competition IV Dataset IIb. It can be seen from Table 12 that CSP-FB+LOG is second only to literature [30]. CSP-WPD+LOG is lower than the literatures [30,84]. The effect of CSP-Wavelet+LOG is relatively poor. Literature [30] uses EMD time-frequency analysis method for feature extraction and CNN for classification. Compared with literature [30], considering the time of feature extraction and the complexity of the model, our method has certain advantages.

In summary, although our method has not achieved the best classification accuracy on each data set, they are better than most existing methods. In addition, our methods have certain advantages in feature extraction time and classification model complexity. Our method uses FLDA for classification, and obviously the complexity of the classification model is relatively low. For the feature extraction time, we will introduce it in detail in the discussion section.

## 4. Discussion

When CSP-Wavelet and CSP-WPD methods are used for feature extraction, the number of wavelet decomposition layers has a greater impact on classification accuracy. The selection for the number of layers considers two factors, namely the frequency resolution and the decomposition time. For a dataset with a sampling rate of 100 Hz, when the number of decomposition layers is less than or equal to 2, the frequency resolution is too low. It is impossible to correctly distinguish the frequency bands related to motor imagery, which is not conducive to extracting discriminative information. When the number of decomposition layers is greater than or equal to 5, the frequency band resolution is too high, and the extracted features are easily affected by noise. At the same time, the decomposition time also increased significantly. Therefore, for a dataset with a sampling rate of 100 Hz (dataset 1), only the case of the number of layers with 3 and 4 are considered in this paper. Similarly, for a dataset with a sampling rate of 250 Hz or 256 Hz (datasets 2–4), we only consider the case of the number of layers with 4 and 5.

Table 13 and Table 14 show the classification results of CSP-Wavelet and CSP-WPD methods using different decomposition layers, respectively. We first discuss the CSP-Wavelet method. In Table 13, when the sampling rate of the dataset is 100 Hz, L_1_ = 3, and L_2_ = 4. When the sampling rate of the dataset is 250 Hz or 256 Hz, L_1_ = 4, and L_2_ = 5. It can be seen from Table 13 that the classification accuracy of the smaller number of decomposition layers is usually greater than that of the larger number of decomposition layers. Even in some cases, the larger number of decomposition layers get a slightly better classification accuracy, considering the decomposition time, we still choose a smaller number of decomposition layers. In Table 14, for CSP-WPD method, we can obtain similar results. Therefore, for the sampling rate of the dataset is 100 Hz, the number of decomposition layer with 3 is selected in this paper. For the sampling rate of the dataset is 250 Hz or 256 Hz, the number of decomposition layer with 4 is selected.

In Table 13 and Table 14, we not only studied the influence of the number of decomposition layers on the classification results, but also studied the influence of sub-band selection on the classification results. As it can be seen from Table 13 and Table 14, in most cases, sub-band selection helps to improve the classification accuracy. Manually excluding sub-bands that are obviously unrelated to the motor imagery tasks can remove redundant information and reduce noise interference, and also reducing feature dimensions and model complexity. Therefore, sub-band selection can improve classification accuracy. It is worth pointing out that, when selecting sub-bands of CSP-Wavelet, the number of decomposition layers has no effect on the classification accuracy. The reason for this is that, when the number of decomposition layers is greater than or equal to three (100 Hz sampling rate) or greater than or equal to four (250 Hz or 256 Hz sampling rate), the selected sub-band are the same.

**Figure 8:**
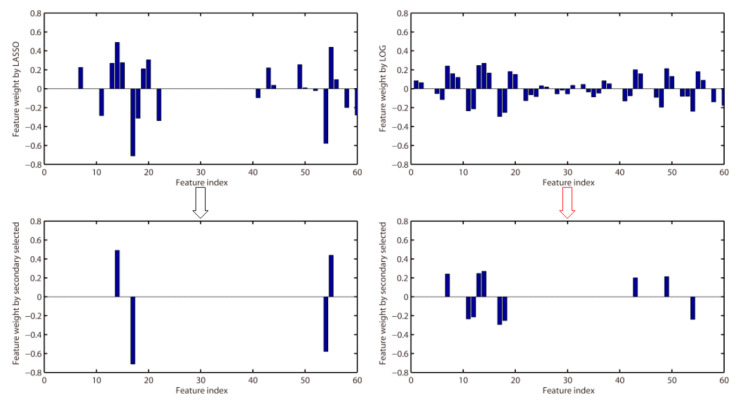
Feature selection by LASSO and LOG for subject A01 with motor imagery tasks of left hand vs right hand.

LASSO has been widely used in EEG feature selection. However, LASSO is a biased estimation of the $l_{0}$-norm, which regularize the feature weights with $l_{1}$-norm. The feature weights obtained by LASSO deviate from the true value and are too sparse. Non-convex regularization can alleviate the bias problem of $l_{1}$-norm [50]. Therefore, the LOG method proposed in this paper can improve the classification accuracy. To illustrate the problem more intuitively, for subject A01 with motor imagery tasks of left hand vs. right hand, Figure 8 shows the feature weights obtained by LASSO and LOG, where features are extracted by CSP-FB. The lower part of Figure 8 is the feature weight obtained by performing the second feature selection on the feature weights obtained by LASSO and LOG. A total of six channels of signals are retained after CSP filtered. The feature index 1–10 in Figure 8 corresponds to the features of the first channel signal after filtered by 8–12 Hz, 10–14 Hz, ..., 26–30 Hz band-pass filters. The feature index 11–20 corresponds to the features of the second channel signal after filtered by 8–12 Hz, 10–14 Hz, ..., 26–30 Hz band-pass filters. The other feature indexes can be deduced by analogy. It can be seen from Figure 8 that the features selected by the LOG method include the features of the first and second channel signals and the fifth and sixth channel signals, while LASSO only selects the features of the second channel signal and the sixth channel signal. Therefore, the features selected by the LOG method contain more information, which is more discriminative (according to the CSP principle, the signals of the front and back m channel signals are more discriminative). In summary, the features selected by LASSO are too few, and at the same time the selected features are not discriminative enough.

**Figure 9:**
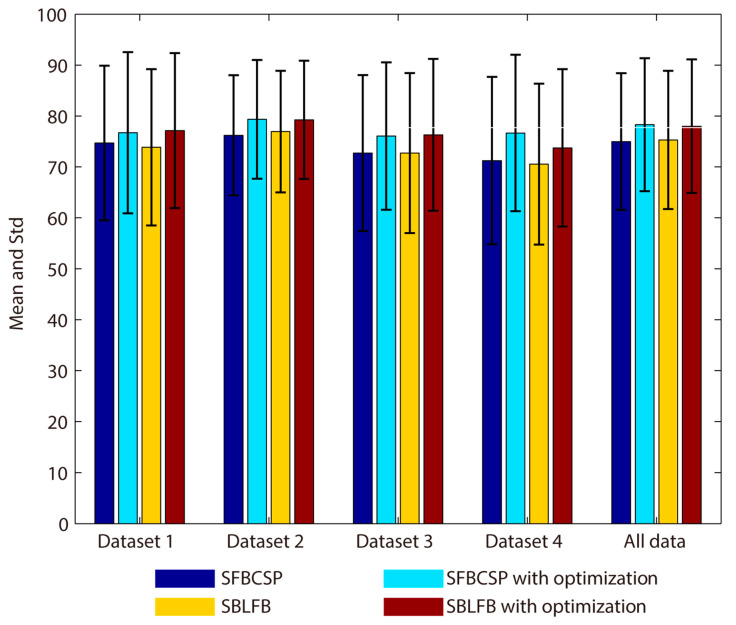
SFBCSP and SBLFB optimized with secondary feature selection (All data).

In the classification results of all datasets, the SFBCSP and SBLFB methods are relatively poor. We use the ensemble learning method proposed in this paper to optimize these two methods, and the results are shown in Figure 9. Although the classification accuracy of the SFBCSP and SBLFB methods are effectively improved, compared with the other methods, the effect is still not good. The SFBCSP and SBLFB methods do not achieve good classification results in this paper, there may be two reasons. On the one hand, the datasets used in this paper are different from the compared methods. The SFBCSP and SBLFB methods may not be applicable to new datasets. On the other hand, although we have tried to restore the SFBCSP and SBLFB methods described by the author, there may be some data processing steps and some details may not be handled properly. It is worth noting that the effect of the algorithms restored in this paper is similar to that in the literature [26]. Specifically, the SFBCSP and SBLFB methods do not perform well on the dataset 1.

Table 15 shows the feature extraction time of training set for each method. Three subjects were used for the experiment, namely 1a, A01, and S01. These three subjects were from three different datasets, and these three datasets had different sampling rates and channel number. The feature extraction process of SFBCSP and SBLFB is the same, so the feature extraction time is the same. Comparing the three proposed methods with existing methods (CSP-Wavelet vs. Wavelet-CSP, CSP-WPD vs. WPD-CSP, and CSP-FB vs. SFBCSP), the feature extraction time is significantly reduced. Among the three newly proposed methods, CSP-FB has the least time. Although CSP-FB takes longer time than CSP, it can still be used in real-time BCI.

The three proposed methods in this paper do not consider the selection of time window during feature extraction. The correct selection of time window can effectively improve the classification accuracy, which has been verified in many existing works, such as literature [44,46]. Therefore, in future work, we will consider integrating the selection of time windows into the proposed methods to further improve classification performance. In addition, the feature selection method proposed in this paper uses cross-validation to obtain model parameters. The model training is cumbersome and time-consuming. On the other hand, the parameters obtained by cross-validation are not necessarily optimal, especially in the case of small samples [85]. Implementing LASSO and LOG under the Bayesian framework [86] to avoid tedious cross-validation will further improve the performance of the proposed methods.

## 5. Conclusions

In this paper, we have proposed three new feature extraction methods and one feature selection method. FLDA is used for classification. Combining feature extraction and feature selection methods, we can obtain three new EEG decoding methods, namely CSP-Wavelet+LOG, CSP-WPD+LOG, and CSP-FB. The classification performance of the proposed methods is better than the existing methods. CSP-WPD+LOG has achieved the highest total average classification accuracy among the three new methods, but the feature extraction time is the longest. The classification accuracy of CSP-Wavelet+LOG and CSP-FB is slightly lower than CSP-WPD+LOG, but these two methods have a huge time advantage, especially CSP-FB, which can be used in real-time brain-computer interface.

In future work, we will continue to optimize the proposed method. For example, we can improve the selection of the optimal filtering frequency band and time window, and the selection method of model parameters. In addition, multi-classification expansion will also be part of our future research content.

## Figures and Tables

**Table 1:** Compared methods.

Methods	Algorithm Composition and Processing Flow
CSP	Band-pass filtered EEG signals are spatially filtered by CSP. The logarithm of the variances of spatially filtered signals are extracted as features [10].
Wavelet-CSP	The EEG signals of each channel are decomposed using DWT, the wavelet base is db4. The number of decomposition layers of dataset 1 is 3, and the other datasets are 4. The sub-bands related to motor imagery are used to reconstruct new channels, and then feature extraction is performed using CSP [13].
WPD-CSP	The EEG signals of each channel are decomposed using WPD, the wavelet base is db4. The number of decomposition layers of dataset 1 is 3, and the other datasets are 4. The sub-bands related to motor imagery are used to reconstruct new channels, and then feature extraction is performed using CSP [15].
SFBCSP	The original EEG signals are filtered into 17 sub-bands, and features are extracted for each sub-band using CSP. The filter bandwidth is 4 Hz and the overlap rate is 2 Hz in the range of 4-40 Hz. The sub-band features are selected by LASSO [23].
SBLFB	The original EEG signals are filtered into 17 sub-bands, and features are extracted for each sub-band using CSP. The filter bandwidth is 4 Hz and the overlap rate is 2 Hz in the range of 4–40 Hz. The sub-band features are selected by sparse Bayesian learning [24].
CSP-Wavelet+LASSO	After band-pass filtering, features are extracted using CSP-Wavelet. Features are selected by LASSO. Ensemble learning is used for secondary feature selection and classification model construction.
CSP-WPD+LASSO	After band-pass filtering, features are extracted using CSP-WPD. Features are selected by LASSO. Ensemble learning is used for secondary feature selection and classification model construction.
CSP-FB+LASSO	After band-pass filtering, features are extracted using CSP-FB. Features are selected by LASSO. Ensemble learning is used for secondary feature selection and classification model construction.
CSP-Wavelet+LOG	After band-pass filtering, features are extracted using CSP-Wavelet. Features are selected by LOG. Ensemble learning is used for secondary feature selection and classification model construction.
CSP-WPD+LOG	After band-pass filtering, features are extracted using CSP-WPD. Features are selected by LOG. Ensemble learning is used for secondary feature selection and classification model construction.
CSP-FB+LOG	After band-pass filtering, features are extracted using CSP-FB. Features are selected by LOG. Ensemble learning is used for secondary feature selection and classification model construction.

**Table 2:** Classification accuracy (Dataset 1).

Subjects	CSP	Wavelet-CSP	WPD-CSP	SFBCSP	SBLFB	CSP-Wavelet+LOG	CSP-WPD+LOG	CSP-FB+LOG
1a	77.00	61.00	59.00	63.00	61.00	67.00	64.00	73.00
1b	67.00	63.00	64.00	54.00	53.00	69.00	69.00	75.00
1c	74.00	80.00	81.00	58.00	58.00	76.00	80.00	87.00
1d	92.00	93.00	92.00	90.00	89.00	97.00	95.00	98.00
1e	97.00	97.00	97.00	96.00	96.00	97.00	100.00	99.00
1f	89.00	86.00	86.00	82.00	80.00	90.00	93.00	93.00
1g	94.00	95.00	72.00	80.00	80.00	94.00	94.00	97.00
Mean ± Std	84.29 ± 10.66	82.14 ± 13.82	78.71 ± 13.20	74.71 ± 15.18	73.86 ± 15.34	84.29 ± 12.26	85.00 ± 13.05	88.86 ± 10.12

**Table 3:** Classification accuracy (Dataset 2).

Subjects	CSP	Wavelet-CSP	WPD-CSP	SFBCSP	SBLFB	CSP-Wavelet+LOG	CSP-WPD+LOG	CSP-FB+LOG
A01-LR	89.58	88.19	90.28	76.39	79.17	93.06	91.67	90.28
A02-LR	56.25	51.39	54.17	52.78	52.78	61.81	55.56	61.81
A03-LR	96.53	93.06	93.75	87.5	88.89	95.83	97.22	97.92
A04-LR	71.53	66.67	64.58	63.89	63.19	72.92	72.22	69.44
A05-LR	52.08	50	54.86	81.94	81.25	58.33	62.5	52.78
A06-LR	70.14	61.81	70.83	57.64	59.03	68.06	68.06	69.44
A07-LR	81.94	82.64	85.42	77.78	82.64	81.25	79.17	77.08
A08-LR	93.06	93.75	94.44	88.19	90.28	95.14	95.14	95.14
A09-LR	89.58	90.28	90.28	85.42	84.03	93.06	93.06	90.97
A01-LF	95.83	95.14	98.61	89.58	90.28	99.31	99.31	97.22
A02-LF	71.53	68.06	72.22	70.83	73.61	69.44	67.36	75.69
A03-LF	94.44	95.83	95.14	90.28	90.97	95.83	95.14	95.83
A04-LF	78.47	83.33	84.72	81.25	80.56	81.94	88.89	85.42
A05-LF	62.5	68.75	54.86	53.47	54.17	71.53	70.83	63.89
A06-LF	66.67	68.06	72.22	64.58	65.28	70.83	68.75	68.75
A07-LF	98.61	99.31	95.83	92.36	94.44	99.31	99.31	100
A08-LF	75.69	83.33	75.69	77.08	78.47	86.11	79.86	84.72
A09-LF	95.83	93.06	93.75	93.75	93.06	97.22	97.22	93.75
A01-LT	95.14	97.92	95.14	88.19	86.81	99.31	98.61	94.44
A02-LT	65.28	61.81	64.58	61.11	62.5	69.44	67.36	63.19
A03-LT	94.44	96.53	96.53	83.33	85.42	95.83	96.53	95.83
A04-LT	87.5	89.58	88.19	76.39	77.78	88.89	88.19	89.58
A05-LT	71.53	67.36	69.44	68.06	70.83	70.14	70.83	72.92
A06-LT	70.14	65.97	71.53	66.67	68.75	70.14	70.14	76.39
A07-LT	98.61	97.22	95.14	88.89	88.89	95.14	96.53	96.53
A08-LT	91.67	89.58	90.97	80.56	84.72	93.75	93.75	88.19
A09-LT	95.83	95.83	96.53	95.83	95.83	97.92	97.92	97.92
A01-RF	93.06	96.53	96.53	77.08	78.47	98.61	98.61	98.61
A02-RF	79.86	68.06	72.92	67.36	67.36	81.94	81.25	77.08
A03-RF	93.06	95.14	95.14	84.03	84.72	93.75	95.83	97.22
A04-RF	89.58	92.36	86.11	81.94	79.86	90.28	93.75	90.28
A05-RF	52.78	55.56	58.33	59.72	58.33	64.58	67.36	63.89
A06-RF	61.81	66.67	67.36	71.53	67.36	66.67	66.67	64.58
A07-RF	97.22	100	98.61	97.22	98.61	100	97.92	98.61
A08-RF	79.86	77.08	71.53	73.61	76.39	77.08	74.31	81.25
A09-RF	83.33	84.03	85.42	74.31	75	86.81	86.81	87.5
A01-RT	98.61	100	100	87.5	85.42	100	100	99.31
A02-RT	67.36	66.67	65.28	61.81	60.42	65.28	65.28	70.83
A03-RT	90.97	96.53	95.83	90.97	89.58	96.53	96.53	96.53
A04-RT	85.42	86.11	84.72	81.94	83.33	85.42	86.81	90.28
A05-RT	57.64	57.64	62.5	81.25	80.56	73.61	74.31	63.89
A06-RT	65.28	68.75	64.58	56.94	58.33	73.61	74.31	67.36
A07-RT	97.92	95.83	97.92	95.14	97.22	99.31	97.92	97.92
A08-RT	90.28	89.58	88.89	78.47	79.86	89.58	91.67	88.89
A09-RT	90.28	86.11	84.03	78.47	82.64	93.75	93.06	80.56
A01-FT	68.75	75.69	73.61	71.53	72.22	74.31	72.22	74.31
A02-FT	70.14	66.67	75	63.89	63.89	78.47	75	70.14
A03-FT	69.44	80.56	78.47	72.92	70.83	75.69	75.69	87.5
A07-FT	59.72	72.22	69.44	75	77.78	71.53	61.11	70.83
A08-FT	60.42	55.56	61.11	50.69	50.69	65.28	59.72	54.86
A09-FT	68.75	72.22	70.14	63.19	65.97	69.44	71.53	70.14
A07-FT	80.56	78.47	80.56	75	74.31	83.33	84.72	78.47
A08-FT	83.33	79.17	82.64	77.08	79.86	82.64	86.81	84.72
A09-FT	93.75	90.28	90.28	72.92	72.22	94.44	94.44	85.42
Mean ± Std	80.36 ± 3.98	80.52 ± 4.36	80.86 ± 3.54	76.21 ± 1.80	76.94 ± 11.94	83.40 ± 12.47	83.05 ± 3.15	82.15 ± 3.16

**Table 4:** Classification accuracy (Dataset 3).

Subjects	CSP	Wavelet-CSP	WPD-CSP	SFBCSP	SBLFB	CSP-Wavelet+LOG	CSP-WPD+LOG	CSP-FB+LOG
B01	77.5	78.75	76.25	71.25	78.75	78.75	81.25	88.75
B02	60	52.5	56.25	46.25	45	56.25	57.5	52.5
B03	46.25	43.75	45	47.5	48.75	45	45	48.75
B04	98.75	98.75	98.75	100	98.75	96.25	96.25	98.75
B05	88.75	91.25	91.25	83.75	76.25	91.25	91.25	88.75
B06	81.25	81.25	75	67.5	63.75	77.5	80	90
B07	81.25	78.75	78.75	85	83.75	85	86.25	90
B08	93.75	93.75	93.75	75	71.25	92.5	92.5	92.5
B09	81.25	81.25	73.75	65	68.75	81.25	78.75	83.75
Mean ± Std	78.75 ± 15.47	77.78 ± 17.32	76.53 ± 16.42	71.25 ± 16.43	70.56 ± 15.8	78.19 ± 16.13	78.75 ± 16.01	81.53 ± 16.95

**Table 5:** Classification accuracy (Dataset 4).

Subjects	CSP	Wavelet-CSP	WPD-CSP	SFBCSP	SBLFB	CSP-Wavelet+LOG	CSP-WPD+LOG	CSP-FB+LOG
S01	56.67	58.33	65.00	63.33	66.67	68.33	70.00	66.67
S02	85.00	90.00	86.67	76.67	78.33	90.00	93.33	90.00
S03	100.00	98.33	100.00	98.33	98.33	100.00	100.00	100.00
S04	85.00	85.00	85.00	76.67	83.33	91.67	91.67	86.67
S05	60.00	58.33	60.00	50.00	50.00	63.33	63.33	71.67
S06	66.67	75.00	66.67	60.00	56.67	76.67	75.00	81.67
S07	90.00	93.33	88.33	93.33	91.67	91.67	91.67	85.00
S08	81.67	83.33	86.67	81.67	81.67	88.33	90.00	91.67
S09	98.33	98.33	98.33	95.00	93.33	100.00	100.00	98.33
S10	53.33	55.00	60.00	51.67	55.00	53.33	60.00	65.00
S11	76.67	80.00	71.67	80.00	80.00	81.67	81.67	80.00
S12	81.67	75.00	75.00	71.67	70.00	88.33	90.00	88.33
S13	60.00	50.00	55.00	61.67	63.33	58.33	61.67	60.00
S14	51.67	70.00	61.67	58.33	50.00	60.00	63.33	56.67
Mean ± Std	74.76 ± 15.93	76.43 ± 15.63	75.72 ± 14.44	72.74 ± 15.31	72.74 ± 15.70	79.40 ± 15.39	80.83 ± 14.29	80.12 ± 13.44

**Table 6:** The effect of secondary feature selection on average classification accuracy of various datasets (the LASSO method).

Datasets	Without Secondary Feature Selection			With Secondary Feature Selection		
	CSP-Wavelet+LASSO	CSP-WPD+LASSO	CSP-FB+LASSO	CSP-Wavelet+LASSO	CSP-WPD+LASSO	CSP-FB+LASSO
dataset 1	80.71	81.86	78.29	82.71	85.43	86
dataset 2	80.53	80.95	79.51	82.69	82.91	82.57
dataset 3	76.94	75.14	77.36	77.64	75.97	80.97
dataset 4	76.55	76.79	73.33	78.57	79.17	78.81
All data	79.5	79.71	78.15	81.46	81.75	82.06

**Table 7:** The effect of secondary feature selection on average classification accuracy of various datasets (the LOG method).

Datasets	Without Secondary Feature Selection			With Secondary Feature Selection		
	CSP-Wavelet+LOG	CSP-WPD+LOG	CSP-FB+LOG	CSP-Wavelet+LOG	CSP-WPD+LOG	CSP-FB+LOG
dataset 1	80.71	81.57	81.71	84.29	85	88.86
dataset 2	80.63	80.4	79.22	83.4	83.05	82.15
dataset 3	75.97	76.11	79.17	78.19	78.75	81.53
dataset 4	77.38	77.86	74.76	79.4	80.83	80.12
All data	79.6	79.62	78.68	82.25	82.38	82.3

**Table 8:** The effect of different feature selection methods on average classification accuracy of various datasets.

Feature Extraction	Feature Selection	Dataset 1	Dataset 2	Dataset 3	Dataset 4	All Data
CSP-Wavelet	F-score	82.86	80.79	75.83	76.79	79.76
	GA	79.29	80.09	75.97	75.12	78.76
	BPSO	81.29	80.17	78.47	76.43	79.46
	LASSO	82.71	82.69	77.64	78.57	81.46
	LOG	84.29	83.4	78.19	79.4	82.25
CSP-WPD	F-score	81.57	80.56	75.56	77.97	79.67
	GA	81.57	80.59	74.72	77.14	79.47
	BPSO	81.14	80.08	74.86	75.59	78.86
	LASSO	85.43	82.91	75.97	79.17	81.75
	LOG	85	83.05	78.75	80.83	82.38
CSP-FB	F-score	82.29	80.74	78.75	77.26	80.07
	GA	81.86	80.03	78.75	74.05	79.05
	BPSO	81.86	79.49	79.17	73.81	78.7
	LASSO	86	82.57	80.97	78.81	82.06
	LOG	88.86	82.15	81.53	80.12	82.3

**Table 9:** The effect of different classifiers on average classification accuracy of various datasets.

Datasets	CSP-Wavelet+LOG			CSP-WPD+LOG			CSP-FB+LOG		
	SVM	BLDA	FLDA	SVM	BLDA	FLDA	SVM	BLDA	FLDA
dataset 1	85	83.14	84.29	84.29	84.29	85	89.14	89.29	88.86
dataset 2	82.11	82.99	83.4	82.82	82.91	83.05	82.54	82.64	82.15
dataset 3	78.47	78.33	78.19	78.75	80.28	78.75	80.83	80.28	81.53
dataset 4	76.67	79.52	79.4	77.02	79.52	80.83	79.52	79.4	80.12
All data	81.06	81.92	82.25	81.54	82.18	82.38	82.4	82.4	82.3

**Table 10:** Classification accuracy of the proposed methods and other resent methods for BCI Competition IV Dataset I.

Methods	1a	1b	1c	1d	1e	1f	1g	Mean ± Std
PELM [70] (2018)	79.00	56.50	59.50	73.00	71.50	64.50	85.00	70.00 ± 10.33
LRFCSP [71] (2019)	87.40	70.00	67.40	92.90	93.40	88.80	93.20	84.70 ± 11.22
OPTICAL [72] (2019)	87.32	61.67	71.83	88.17	89.00	85.83	93.83	82.53 ± 8.17
BF [73] (2020)	86.24	88.31	92.89	89.51	90.92	88.46	90.16	89.50 ± 2.12
SCSP-RDA [74] (2020)	97.00	96.00	72.50	75.00	78.50	96.00	95.50	87.21 ± 11.26
CSP-Wavelet+LOG	67	69	76	97	97	90	94	84.29 ± 12.26
CSP-WPD+LOG	64	69	80	95	100	93	94	85.00 ± 13.05
CSP-FB+LOG	73	75	87	98	99	93	97	88.86 ± 10.12

**Table 11:** Classification accuracy of the proposed methods and other resent methods for BCI Competition IV Dataset IIa.

Methods	A01	A02	A03	A04	A05	A06	A07	A08	A09	Mean ± Std
GRU-RNN [75] (2018)	84.82	65.32	83.54	67.67	64.00	70.87	84.96	71.95	68.90	73.56 ± 4.38
CSP\AM-BA-SVM [76] (2018)	90.56	66.32	91.99	70.28	68.53	55.75	90.63	87.80	85.07	78.55 ± 13.40
ERDSA [77] (2018)	86.81	63.89	94.44	68.75	56.25	69.44	78.47	97.91	93.75	78.86 ± 15.07
IST-TSVM [78] (2019)	80.14	51.55	95.54	53.60	51.65	56.83	56.58	93.42	92.66	70.22 ± 19.74
CA+PSR+CSP [79] (2020)	80.00	65.36	87.14	67.50	55.54	50.18	91.79	84.11	87.86	74.39 ± 15.18
MTFL [80] (2020)	91.67	63.19	95.14	72.22	64.58	68.06	79.17	97.92	92.37	80.48 ± 13.97
p-LTCSP [81] (2020)	82.60	70.23	70.23	55.15	54.36	60.14	73.38	85.29	74.62	69.56 ± 11.10
CSP-Wavelet+LOG	93.06	61.81	95.83	72.92	58.33	68.06	81.25	95.14	93.06	79.91 ± 15.06
CSP-WPD+LOG	91.67	55.56	97.22	72.22	62.50	68.06	79.17	95.14	93.06	79.40 ± 15.56
CSP-FB+LOG	90.28	61.81	97.92	69.44	52.78	69.44	77.08	95.14	90.97	78.32 ± 16.02

**Table 12:** Classification accuracy of the proposed methods and other resent methods for BCI Competition IV Dataset IIb.

Methods	B01	B02	B03	B04	B05	B06	B07	B08	B09	Mean ± Std
DBN [82] (2018)	70.38	70.34	71.20	71.24	71.21	70.52	70.79	70.49	70.32	70.72 ± 0.40
CapsNet [83] (2019)	78.75	55.71	55.00	95.93	83.12	83.43	75.62	91.25	87.18	78.44 ± 14.44
SGRM [48] (2019)	77.30	59.10	51.50	97.00	87.40	72.50	86.70	84.70	85.60	78.00 ± 2.30
NCFS [84] (2020)	79.25	63.48	56.65	99.28	88.67	79.96	88.76	92.66	84.95	81.52 ± 13.72
CEMD [30] (2020)	80.56	65.44	65.97	99.32	89.19	86.11	81.25	88.82	86.81	82.61 ± 11.00
CSP-Wavelet+LOG	78.75	56.25	45	96.25	91.25	77.5	85	92.5	81.25	78.19 ± 16.13
CSP-WPD+LOG	81.25	57.5	45	96.25	91.25	80	86.25	92.5	78.75	78.75 ± 16.01
CSP-FB+LOG	88.75	52.5	48.75	98.75	88.75	90	90	92.5	83.75	81.53 ± 16.95

**Table 13:** The effect of wavelet decomposition layers and sub-band selection on average classification accuracy of various datasets (the CSP-Wavelet method).

Dataset	Without Sub-Bands Selected		With Sub-Bands Selected	
	CSP-Wavelet+LOG (L1)	CSP-Wavelet+LOG (L2)	CSP-Wavelet+LOG (L1)	CSP-Wavelet+LOG (L2)
dataset 1	83	82.86	84.29	84.29
dataset 2	83.03	82.96	83.4	83.4
dataset 3	79.72	79.44	78.19	78.19
dataset 4	80.12	80.48	79.4	79.4
All data	82.19	82.16	82.25	82.25

**Table 14:** The effect of wavelet decomposition layers and sub-band selection on average classification accuracy of various datasets (the CSP-WPD method).

Dataset	Without Sub-Bands Selected		With Sub-Bands Selected	
	CSP-WPD+LOG (L1)	CSP-WPD+LOG (L2)	CSP-WPD+LOG (L1)	CSP-WPD+LOG (L2)
dataset 1	84.14	83.86	85	84.71
dataset 2	82.65	80.95	83.05	82.51
dataset 3	78.33	76.81	78.75	77.36
dataset 4	77.62	77.86	80.83	80.48
All data	81.47	80.24	82.38	81.8

**Table 15:** Feature extraction time of training set for each method (Unit: second).

Subject	CSP	Wavelet-CSP	WPD-CSP	SFBCSP (SBLFB)	CSP-Wavelet	CSP-WPD	CSP-FB
1a	0.145	11.702	60.797	1.037	2.109	8.299	0.543
A01	0.126	8.420	36.241	0.850	3.648	19.525	0.809
S01	0.096	3.286	17.283	0.646	2.650	13.636	0.492
